# The Moderating Role of Psychological Flexibility on the Association between Distress-Driven Impulsivity and Problematic Internet Use

**DOI:** 10.3390/ijerph19159592

**Published:** 2022-08-04

**Authors:** Chang Liu, Kristian Rotaru, Samuel R. Chamberlain, Lei Ren, Leonardo F. Fontenelle, Rico S. C. Lee, Chao Suo, Kavya Raj, Murat Yücel, Lucy Albertella

**Affiliations:** 1 BrainPark, Turner Institute for Brain and Mental Health and School of Psychological Sciences, Monash University, Clayton, VIC 3168, Australia; 2 Monash Business School, Monash University, Caulfield, VIC 3145, Australia; 3 Department of Psychiatry, Faculty of Medicine, University of Southampton, Southampton SO17 1BJ, UK; 4 Southern Health NHS Foundation Trust, Southampton SO40 2RZ, UK; 5 Military Medical Psychology School, Fourth Military Medical University, Xi’an 710032, China; 6 D’Or Institute & Institute of Psychiatry, Federal University of Rio de Janeiro, Rio de Janeiro 22290-140, Brazil

**Keywords:** psychological inflexibility, distress-driven impulsivity, problematic internet use, negative urgency, compulsivity

## Abstract

Background: Problematic internet use is receiving increasing attention in the addiction field, yet the mechanisms driving such behaviours remain unclear. Previous research has shown that impulsivity- and compulsivity-related constructs may interactively contribute to a range of problematic behaviours. The current study examined whether distress-driven impulsivity and psychological flexibility may interactively contribute to problematic internet use, which has not been addressed in prior literature. Method: Two hundred and one participants completed an online survey. Bootstrapped moderation analysis was conducted to examine the collected data on distress-driven impulsivity, psychological flexibility, and their interaction in relation to problematic internet use. Results: The interaction between distress-driven impulsivity and psychological flexibility was significantly related to problematic internet use. Simple slope tests confirmed that distress-driven impulsivity was associated with problematic internet use among individuals with low flexibility levels. Conclusions: Our findings highlight the moderating role of psychological inflexibility in the association between distress-driven impulsivity and problematic internet use. Prevention and/or early interventions for problematic internet use should consider targeting psychological inflexibility and distress-driven impulsivity.

## 1. Introduction

Problematic internet use (PIU; loss of control over internet use which leads to negative consequences in daily life, Ref. [1]) has been recognized as a public health concern by 31 nations (i.e., estimated prevalence rate = 7.02%; Ref. [2]). University students, most of whom were born in the internet age and brought up in an environment with easy access to the internet, may be particularly vulnerable to PIU [3]. According to a large-scale (*n* = 2794) international study, the overall prevalence of PIU among university students was 8.4% [4]. PIU has been associated with both physical and psychological concerns, including insomnia, eye strain, psychological distress and elevated suicidality [5,6,7]. Given the high prevalence of PIU and its associated health concerns, it is important to understand factors that drive PIU.

One candidate construct for understanding PIU is distress-driven impulsivity (DDI; the tendency to act rashly in response to negative emotions, Ref. [8]). Individuals characterised by DDI may be more vulnerable to negative emotions and tend to engage in impulsive behaviours under emotional duress [9]. Such a tendency may increase their likelihood of developing various maladaptive behaviours, including PIU [10,11,12,13]. Within the multidimensional impulsivity construct (i.e., comprised of lack of conscientious, sensation seeking and emotion-related impulsivity; Ref. [14]), DDI is the only facet (compared to other facets of impulsivity) that has been identified as a common risk factor across problematic behaviours (e.g., problematic alcohol use, eating and self-harm; [10,13]). In relation to PIU, DDI is the only impulsivity-related trait that is consistently associated with both general PIU [11,12] and specific online activities such as video games, Facebook and pornography [15,16,17].

The European Cooperation in Science and Technology (COST) Action Programme has proposed that, in addition to impulsivity-related constructs, compulsivity-related constructs should also be incorporated to advance understanding of PIU [18]. Compulsivity can be defined as the tendency to engage in repetitive, habitual behaviours that are difficult to control or interfere with current goals [19,20]. One compulsivity-related construct pertinent to PIU is psychological inflexibility/rigidity. In the current study, psychological inflexibility is defined as the inability to adapt mindset or behaviour according to situational demands [21]. Individuals characterised by inflexibility may be more likely to behave in habitual patterns, such as repetitive internet use, despite negative consequences, which may increase their likelihood of developing PIU [22]. The positive relationship between inflexibility and PIU has been reported in neuroimaging research using behavioural measures [23,24,25]. Few studies have examined the inflexibility–PIU association using self-report measures (e.g., Refs. [26,27]). Findings from these studies have shown that psychological inflexibility is associated with greater PIU [26,27].

Existing research has demonstrated the independent role of DDI and inflexibility in relation to PIU. Yet, there has been a lack of studies investigating whether DDI and inflexibility may interact to drive PIU. Empirical evidence suggests that impulsivity- and compulsivity-related constructs may interact to drive greater risk across problematic behaviours, including obsessive compulsive behaviours, problematic drinking and problematic eating [13,28]. More precisely, previous studies have found that inflexibility at the cognitive level (indexed by the persistence of Pavlovian conditioned behaviour following reversal) may moderate the relationship between impulsivity and problematic behaviours [13,28]. The interaction effect between impulsivity and inflexibility on PIU can be explained as follows: individuals characterised by DDI may be vulnerable to distress and tend to react rashly in response to negative emotions. This may predispose them to engage in behaviours that provide instant, short-term relief (e.g., internet use; Refs. [29,30]). This propensity increases the likelihood that individuals may learn to pair internet use with feelings of stress reduction, reinforcing its use (via negative reinforcement) as a coping strategy [29,31]. Despite the short-term relief provided by internet use, such behaviours may induce long-term negative consequences (e.g., failing to fulfil school or professional obligations or abandoning hobbies due to time spent online; Ref. [32]). When facing these negative consequences, flexible individuals may change their behaviours and choose alternative/more adaptive coping strategies that suit current needs. Meanwhile, inflexible individuals may persist in using the internet regardless of the consequences.

Despite the mechanistic insights that may be gained from the proposed interaction and its potential for informing early detection of and interventions for individuals at risk for PIU, no study has examined whether DDI and psychological flexibility interact in the context of PIU. Further, it is unclear whether the moderating role of flexibility (at the cognitive level) may be replicated at the trait level. To address the aforementioned questions, the current study examined how DDI and psychological flexibility may interact and drive PIU. We hypothesised that psychological flexibility levels may moderate the relationship between DDI and PIU. More precisely, DDI will correlate to PIU among individuals characterised by inflexibility, whereas no such relationship will be seen among individuals with high flexibility levels.

## 2. Method

### 2.1. Participants

**Figure 1:**
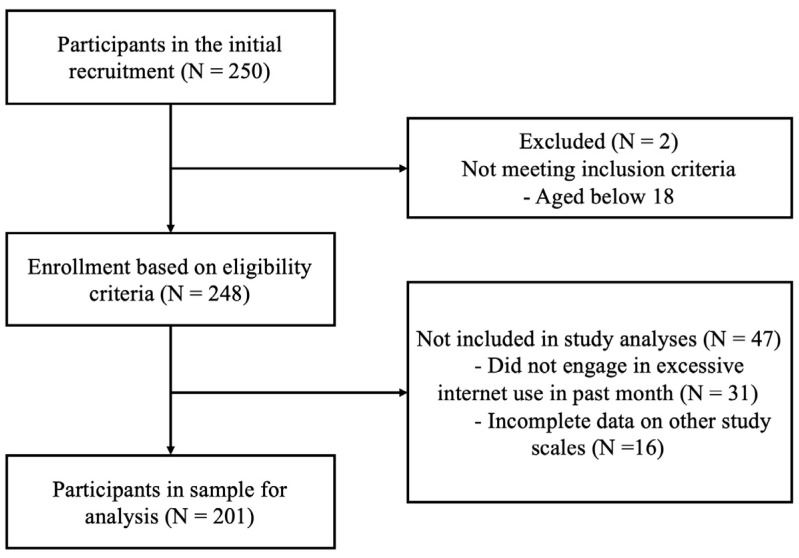
The flow chart of the study procedures.

The current study adopted a convenience sampling method for participant recruitment. Participants were recruited through a student research participation pool at Monash University. Participants received bonus credit in their course for participating in the study as an incentive. Participation was voluntary, and the same bonus credit was applied for participating in any research study offered to the pool. All study measures were delivered via Qualtrics as an online survey. Only adult participants (aged 18 and above) who provided informed consent were included in the study. Participants who reported excessive internet use in the past month were included for data analysis. The final sample consisted of 201 participants. The flow chart of the study procedure is presented in Figure 1.

### 2.2. Ethics

All participants consented prior to participating in the study. The study procedures followed the Declaration of Helsinki and was approved by the Human Research Ethics Committee (Project number 24091).

### 2.3. Measurements

Demographic related questions were asked at the beginning of the survey, and participants completed the following measures:

Short version of the Urgency, Premeditation (lack of), Perseverance (lack of), Sensation Seeking, and Positive Urgency Impulsivity Behaviour Scale (S-UPPS-P; Ref. [14]): This is a 20-item scale examining trait impulsivity. There are five subscales covering: Negative Urgency (e.g., “When I feel bad, I will often do things I later regret in order to make myself feel better now”), Positive Urgency (the tendency to react rashly under positive emotions), Lack of Perseverance (the inability to focus on tasks), Lack of Premeditation (the tendency to act without thinking of consequences) and Sensation Seeking (the tendency to pursue thrilling experiences). Each item was rated on a four-point Likert scale ranging from 1 (strongly agree) to 4 (strongly disagree). The Negative Urgency subscale total score (reverse coded) was used to measure DDI. In the current study, the Negative Urgency subscale showed acceptable reliability (Cronbach’s alpha = 0.71). 

**Psychological Flexibility Measure:** We adopted the General Flexibility Subscale from the Eating Disorder Flexibility Index Questionnaire (EDFLIX, Ref. [33]) to measure psychological flexibility levels. The subscale consists of 17 items, which measures psychological flexibility levels in both cognitive and behavioural aspects. Sample items include “I find it difficult to get used to new situations” and “When things don’t go according to plan, I am able to consider alternative solutions”. Each item was rated on a six-point Likert scale ranging from 1 (strongly disagree) to 6 (strongly agree). The total score (reverse coded, with higher scores indicating greater flexibility) of the General Flexibility Subscale was used to measure psychological flexibility levels. In the current study, the General Flexibility Subscale showed good reliability (Cronbach’s alpha = 0.85).

Brief Assessment Tool of Compulsivity Associated Problems (BATCAP; Ref. [34]): This is a 6-item measurement tool for problematic behaviours that share compulsive features. Individuals who reported having engaged in excessive internet use (using the internet longer than intended, not including work or necessary tasks) in the past month were asked to complete the internet BATCAP. The scale asked participants to report time spent (“On average, how much time was occupied by excessive internet use”), distress (“How much distress did excessive internet use cause you”), loss of control (“How hard was it for you to control excessive internet use”), functional impairments (“How much did excessive internet use interfere with work/school, social, or family life”), anxiety if prevented from using the internet (“How anxious would you become if prevented from excessive internet use”), and strongest urges (“At its most severe point (in the past week), what was the strength of your strongest urge/craving to perform excessive internet use”) over the past week. Each item was rated on a five-point Likert scale ranging from 0 to 4. The BATCAP is considered to reflect the transdiagnostic nature of compulsivity and has been recommended to be used when measuring compulsivity related behaviours [35]. In the context of PIU, it is highly correlated with the Internet Addiction Test (rs = 0.60, *p* < 0.001, Ref. [34]). The total BATCAP internet score was used to index PIU. The BATCAP internet scale showed good reliability in the current study (Cronbach’s alpha = 0.86).

The Depression Anxiety, Stress Scale—Short Form (DASS-21; Ref. [36]): This is a widely used 21-item questionnaire that examines the negative emotional states of depression, anxiety and stress. The total score of DASS-21, which indexes psychological distress, was entered as a covariate. In the current study, the DASS-21 scale showed excellent reliability (Cronbach’s alpha = 0.94).

### 2.4. Data Analysis

**Figure 2:**
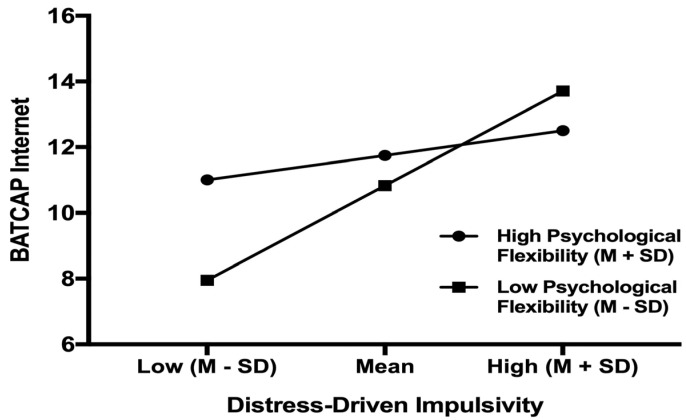
Simple Slope Test. Note: The moderating role of psychological flexibility in the association between DDI and PIU. The moderating effect is graphed for two levels of psychological flexibility: low flexibility/inflexible (1 SD below the mean) and high flexibility/flexible (1 SD above the mean).

We examined descriptive statistics and Spearman correlations across DDI, psychological flexibility and PIU. Expression of N (%) and mean ± SD were used for categorical and continuous data, respectively. A bootstrapped moderation analysis was performed to test the interaction effect of DDI and psychological flexibility on PIU. The analysis was conducted via PROCESS macro for SPSS version 26 (IBM Corp., Armonk, NY, USA), which utilised a bias-corrected bootstrap method. Following Hayes’s recommendation [37], we used 5000 bootstrap samples and mean-centred all continuous variables. DDI was set as the independent variable, psychological flexibility was set as the moderator, and BATCAP internet score was set as the dependent variable. Age, gender, the other four facets of impulsivity, and psychological distress were entered as covariates to avoid confounding effects [34]. Conditional effect analysis and simple slope tests were used to follow up on the significant interaction term. The significance of simple slopes was tested and plotted at low (−1 SD) and high (+1 SD) levels of psychological flexibility (Figure 2).

## 3. Results

The sample included 201 students (130 females), aged 18–36 years (Median = 21.39, SD = 2.52). Age was not associated with PIU (rs (201) = 0.05, *p* = 0.47). Further, Mann–Whitney *U* test showed that PIU scores did not differ by gender U (Nfemale = 130, Nmale = 71) = 4590.00, z = −0.06, *p* = 0.95. Descriptive statistics and correlations across variables were presented in Table 1. A significant interaction effect (β = −0.05, SE = 0.02, 95% CI = [−0.09, −0.01]) between DDI and psychological flexibility score on PIU was found when controlling for age, gender, DASS-21 total score and other impulsivity facets (see Table 2). The interaction effect was followed up by conditional effect analysis. According to the result, DDI was positively associated with PIU among participants with low levels of psychological flexibility (β = 1.33, SE = 0.34, 95% CI = [0.67, 2.00]). In contrast, the effect was not significant among participants with high levels of psychological flexibility (β = 0.34, SE = 0.33, 95% CI = [−0.31, 0.99]). The associations between DDI and PIU at low (−1 SD) and high (+1 SD) levels of psychological flexibility were plotted via simple slope tests (Figure 2).

## 4. Discussion

The current study aimed to examine the interaction effect between DDI and psychological flexibility in relation to PIU. Specifically, we found that DDI and psychological flexibility may interactively contribute to PIU. Further, the positive relationship between DDI and PIU was significant among individuals with low levels of flexibility. Such an association did not exist among people with high levels of flexibility.

The role of DDI in relation to problematic behaviours has been demonstrated extensively in existing research across addictive behaviours, including PIU [11,38]. Similarly, psychological inflexibility has been found to be a risk factor for compulsive behaviours [39,40] as well as PIU [26,27]. Despite DDI and psychological inflexibility’s individual contributions to problematic behaviours, growing evidence supports that the interaction between impulsivity- and compulsivity-related constructs may be a transdiagnostic mechanism underlying various problematic behaviours. As such, the interaction effect warrants attention in its own right.

The current study extends previous research in two important ways. First, like other problematic behaviours (e.g., problematic alcohol use and eating; Refs. [13,28]), we found that PIU may be explained by the interaction between impulsivity- and compulsivity-related constructs. Second, in addition to cognitive inflexibility, we found that psychological inflexibility may also moderate the relationship between impulsivity and problematic behaviours. In other words, this study extends the previous cognitive findings to trait inflexibility, highlighting parallels between cognitive and psychological inflexibility, as well as supporting the use of non-cognitive methods of assessing risk (which have greater potential for scalability). As introduced previously, the interplay between DDI and psychological inflexibility may drive PIU as follows: people high in DDI are more vulnerable to distress and tend to engage in instantly gratifying/soothing behaviours (e.g., internet use) when being confronted with stressful life events and associated emotions. As these behaviours may provide short-term relief to negative mood and thereby, through negative reinforcement, may be acquired as a coping strategy [31]. Over time, such coping strategies may bring aversive consequences (e.g., too much time spent online at the expense of time spent on work, etc.). At this point, flexible individuals may be able to change their behaviour (e.g., use alternative strategies to cope with stress), and thereby avoid negative consequences. However, inflexible individuals may not be able to change their behaviour so easily, and so would be more likely to continue using the internet as a coping strategy, despite its adverse consequences [13,28].

In the current study, age and gender did not emerge as significant predictors of PIU. One study found age and gender to be associated with PIU [41]. Meanwhile, non-significant results have also been reported (e.g., Refs. [42,43]). Compared to participants (mean age = 19.3, SD = 1.1) included in [41], participants in our study are generally older (mean age = 21.39, SD = 2.52). The differences in age brackets indicate that participants may face different stressors, have different purposes for using the internet, and have different levels of inhibitory control due to maturation [44], which could ultimately lead to our current findings. Meanwhile, cultural differences (e.g., interpersonal fear tendencies among Japanese males; [41]) may account for whether gender may impact internet use.

Current findings may contribute to both theoretical understanding and intervention developments in PIU. The COST Action Programme highlights that clarifying the possible role of personality features, promoting early identification of at-risk individuals and generating effective interventions should be prioritised for future PIU research [18]. In response to this initiative, the current study has elucidated the interplay between two personality features (i.e., DDI and inflexibility) in relation to PIU. Further, by extending previous results to trait levels, the findings have important implications for early detections of PIU. Specifically, it is usually time-consuming to administer and score behavioural measures of flexibility. This limitation makes it impractical for practitioners to use such measures as screening tools. On the other hand, the self-report measures used in the current study are quick to administer (less than five minutes in total). Moreover, as such measures are independent of symptoms, they may help practitioners identify the risk of PIU and provide timely support even before the onset of symptoms and related problems.

Given that the onset of PIU may contribute to the long-term societal burden (e.g., productivity loss), developing prevention and/or early interventions for PIU may have important public health implications. Several research priorities have been identified when developing prevention and/or early interventions, including the need for: (1) consistent PIU instruments that are effective in identifying PIU at a subclinical level, (2) evaluating the role of protective factors in preventing/reducing progression of PIU, and (3) complex/transdiagnostic interventions that may benefit PIU as well as other risk behaviours simultaneously [18,45,46]. Our findings may address the aforementioned research priorities in several ways. First, the transdiagnostic scale we used in the current study (i.e., BATCAP) may fulfil the need for a consistent and sensitive transdiagnostic and dimensional PIU measure [18,45,46], as it provides a standardised measurement of symptom severity that may be applied across different types of behaviours (including for different forms of internet use, e.g., pornography, social media, etc.) and employs dimensional ratings of symptoms [34,35]. Further, our results indicate that scientifically rigorous prevention and/or early interventions are required to evaluate the effectiveness of improving both DDI and inflexibility in preventing/reducing progressions of PIU. These programmes may include cognitive training on cognitive drivers of both traits (i.e., inhibitory control and cognitive flexibility) and trait modification. Third, personality-targeted coping skills interventions have demonstrated efficacy in targeting problematic substance use [47]. As such interventions focus on the underlying transdiagnostic process (i.e., helping individuals develop more adaptive coping strategies other than risk behaviours), it is worthwhile for future research to examine whether these interventions may concurrently reduce PIU and other risk behaviours.

The current study has several limitations. First, the findings are not able to be interpreted in terms of the directionality of effects due to the study’s cross-sectional nature. Longitudinal research is needed to confirm whether there is a causal relationship between psychological inflexibility, DDI and PIU. Second, the results were generated in a student sample of volunteers receiving study credit, so further work is needed to examine whether the findings can be generalised to other populations. However, as previously mentioned, PIU was more prevalent among university students, which is a key reason for the current study to use a student sample. Lastly, the current study focused on internet use in general; future research may benefit from examining specific online activities participants engage in to identify potential endophenotypes.

## 5. Conclusions

In summary, the current study demonstrates that psychological flexibility and DDI interact in relation to PIU, with DDI being related to PIU among inflexible individuals. The current findings add to the growing literature highlighting the interaction between impulsivity- and compulsivity-related constructs as a potential driver of risk for problematic behaviours.

The findings highlight the potential for brief trait screeners to identify risk for PIU independently of symptoms as well as informing innovative prevention and/or early interventions that target both DDI and inflexibility to reduce risks for PIU. By recognising the role played by DDI and inflexibility, our results may help generate new prevention and/or early interventions for PIU.

## Figures and Tables

**Table 1:** Descriptive statistics and Spearman’s correlation.

Variable	Mean	SD	1	2	3	4	5	6	7	8	9
1. DDI	9.45	2.21									
2. Psychological Flexibility	64.08	10.44	−0.31 **								
3. PIU	11.68	6.86	0.30 **	−0.18 **							
4. Lack of Premeditation	8.24	1.90	0.10	−0.25 **	0.09						
5. Lack of Perseverance	8.59	1.80	−0.15 *	<0.01	0.12	0.48 **					
6. Positive Urgency	8.77	2.24	0.54 **	−0.21 **	0.12	0.17 *	−0.11				
7. Sensation Seeking	9.79	2.29	0.12	0.20 **	0.11	0.06	−0.15 *	0.35 **			
8. Psychological Distress	16.31	11.89	0.46 **	−0.46 **	0.35 **	0.01	−0.16 *	0.30 **	0.08		
9. Age	21.39	2.52	0.06	0.02	0.05	0.08	0.11	0.16 *	0.07	0.03	

Note. N = 201. DDI (Independent variable; measured using the S-UPPS-P negative urgency subscale); Psychological Flexibility (Moderator; measured using the EDFLIX general flexibility subscale); PIU (Dependent variable: measured using the BATCAP Internet scale); Lack of Premeditation (Covariate; measured using the S-UPPS-P lack of premeditation subscale); Lack of Perseverance (Covariate; measured using the S-UPPS-P lack of perseverance subscale); Positive Urgency (Covariate; measured using the S-UPPS-P positive urgency subscale); Sensation Seeking (Covariate; measured using the S-UPPS-P sensation seeking subscale); Psychological Distress (Covariate; measured using the DASS-21 scale). Detailed definitions of each variable can be found under the Measurements section. * *p* < 0.05, ** *p* < 0.01.

**Table 2:** Results of bootstrapped moderation analysis.

Variable	Beta	BootSE	LLCI	ULCI
DDI	0.78	0.27	0.27	1.34
Psychological Flexibility	−0.03	0.05	−0.03	0.08
Interaction Term	−0.05	0.02	−0.09	−0.01
Lack of Premeditation	0.39	0.31	−0.21	0.99
Lack of Perseverance	0.38	0.33	−0.24	1.04
Positive Urgency	−0.44	0.24	−0.92	0.03
Sensation Seeking	0.35	0.23	−0.09	0.80
Psychological Distress	0.16	0.05	0.06	0.25
Age	0.21	0.18	−0.12	0.59
Gender	−1.43	1.04	−3.56	0.55

Note. BootSE: Bootstrapped standard errors; LLCI and ULCI: 95% bootstrapped confidence intervals. DDI (Independent variable; measured using the S-UPPS-P negative urgency subscale); Psychological Flexibility (Moderator; measured using the EDFLIX general flexibility subscale); Interaction Term (DDI*Psychological Flexibility); Lack of Premeditation (Covariate; measured using the S-UPPS-P lack of premeditation subscale); Lack of Perseverance (Covariate; measured using the S-UPPS-P lack of perseverance subscale); Positive Urgency (Covariate; measured using the S-UPPS-P positive urgency subscale); Sensation Seeking (Covariate; measured using the S-UPPS-P sensation seeking subscale); Psychological Distress (Covariate; measured using the DASS-21 scale). Detailed definitions of each variable can be found under the Measurements section.

## Data Availability

The data presented in this study are available on request from the corresponding author.

## References

[1] Spada M.M. (2014). An overview of problematic Internet use. Addict. Behav..

[2] Pan Y.-C., Chiu Y.-C., Lin Y.-H. (2020). Systematic review and meta-analysis of epidemiology of internet addiction. Neurosci. Biobehav. Rev..

[3] Seki T., Hamazaki K., Natori T., Inadera H. (2019). Relationship between internet addiction and depression among Japanese university students. J. Affect. Disord..

[4] Balhara Y.P.S., Doric A., Stevanovic D., Knez R., Singh S., Chowdhury M.R.R., Kafali H.Y., Sharma P., Vally Z., Vu T.V. (2019). Correlates of Problematic Internet Use among college and university students in eight countries: An international cross-sectional study. Asian J. Psychiatry.

[5] Balhara Y.P.S., Mahapatra A., Sharma P., Bhargava R. (2018). Problematic internet use among students in South-East Asia: Current state of evidence. Indian J. Public Health.

[6] Andrade A.L.M., Scatena A., Bedendo A., Enumo S.R.F., Dellazzana-Zanon L.L., Prebianchi H.B., de Lara Machado W., de Micheli D. (2020). Findings on the relationship between Internet addiction and psychological symptoms in Brazilian adults. Int. J. Psychol..

[7] Stevens C., Zhang E., Cherkerzian S., Chen J.A., Liu C.H. (2020). Problematic internet use/computer gaming among US college students: Prevalence and correlates with mental health symptoms. Depress. Anxiety.

[8] Whiteside S.P., Lynam D.R. (2001). The five factor model and impulsivity: Using a structural model of personality to understand impulsivity. Personal. Individ. Differ..

[9] Cyders M.A., Dzemidzic M., Eiler W.J., Coskunpinar A., Karyadi K.A., Kareken D.A. (2015). Negative urgency mediates the relationship between amygdala and orbitofrontal cortex activation to negative emotional stimuli and general risk-taking. Cereb. Cortex.

[10] Dir A.L., Karyadi K., Cyders M.A. (2013). The uniqueness of negative urgency as a common risk factor for self-harm behaviors, alcohol consumption, and eating problems. Addict. Behav..

[11] Burnay J., Billieux J., Blairy S., Larøi F. (2015). Which psychological factors influence Internet addiction? Evidence through an integrative model. Comput. Hum. Behav..

[12] Liu S.-J., Lan Y., Wu L., Yan W.-S. (2019). Profiles of impulsivity in problematic internet users and cigarette smokers. Front. Psychol..

[13] Albertella L., Le Pelley M.E., Chamberlain S.R., Westbrook F., Lee R.S., Fontenelle L.F., Grant J.E., Segrave R., McTavish E., Yücel M. (2020). Reward-related attentional capture and cognitive inflexibility interact to determine greater severity of compulsivity-related problems. J. Behav. Ther. Exp. Psychiatry.

[14] Cyders M.A., Littlefield A.K., Coffey S., Karyadi K.A. (2014). Examination of a short English version of the UPPS-P Impulsive Behavior Scale. Addict. Behav..

[15] Billieux J., Chanal J., Khazaal Y., Rochat L., Gay P., Zullino D., Van der Linden M. (2011). Psychological predictors of problematic involvement in massively multiplayer online role-playing games: Illustration in a sample of male cybercafé players. Psychopathology.

[16] Rothen S., Briefer J.-F., Deleuze J., Karila L., Andreassen C.S., Achab S., Thorens G., Khazaal Y., Zullino D., Billieux J. (2018). Disentangling the role of users’ preferences and impulsivity traits in problematic Facebook use. PLoS ONE.

[17] Sallie S.N., Ritou V.J., Bowden-Jones H., Voon V. (2021). Assessing online gaming and pornography consumption patterns during COVID-19 isolation using an online survey: Highlighting distinct avenues of problematic internet behavior. Addict. Behav..

[18] Fineberg N.A., Demetrovics Z., Stein D.J., Ioannidis K., Potenza M.N., Grünblatt E., Brand M., Billieux J., Carmi L., King D.L. (2018). Manifesto for a European research network into Problematic Usage of the Internet. Eur. Neuropsychopharmacol..

[19] Luigjes J., Lorenzetti V., de Haan S., Youssef G.J., Murawski C., Sjoerds Z., van den Brink W., Denys D., Fontenelle L.F., Yücel M. (2019). Defining compulsive behavior. Neuropsychol. Rev..

[20] Robbins T. (2014). Current perspectives on compulsivity. Int. J. Neuropsychopharmcol..

[21] Gabrys R.L., Tabri N., Anisman H., Matheson K. (2018). Cognitive control and flexibility in the context of stress and depressive symptoms: The cognitive control and flexibility questionnaire. Front. Psychol..

[22] Chamberlain S.R., Leppink E.W., Redden S.A., Stein D.J., Lochner C., Grant J.E. (2017). Impact of obsessive-compulsive personality disorder (OCPD) symptoms in Internet users. Ann. Clin. Psychiatry.

[23] Dong G., Lin X., Zhou H., Lu Q. (2014). Cognitive flexibility in internet addicts: fMRI evidence from difficult-to-easy and easy-to-difficult switching situations. Addict. Behav..

[24] Kim Y.R., Kim J.E., Kim M.H. (2010). Impaired set-shifting ability in patients with eating disorders, which is not moderated by their catechol-o-methyltransferase val158met genotype. Psychiatry Investig..

[25] Zhou Z., Yuan G., Yao J. (2012). Cognitive biases toward Internet game-related pictures and executive deficits in individuals with an Internet game addiction. PLoS ONE.

[26] Jiang Q., Leung L. (2012). Effects of individual differences, awareness-knowledge, and acceptance of Internet addiction as a health risk on willingness to change Internet habits. Soc. Sci. Comput. Rev..

[27] Sagar M.E. (2021). Predictive Role of Cognitive Flexibility and Self-Control on Social Media Addiction in University Students. Int. Educ. Stud..

[28] Liu C., Rotaru K., Lee R.S., Tiego J., Suo C., Yücel M., Albertella L. (2021). Distress-driven impulsivity interacts with cognitive inflexibility to determine addiction-like eating. J. Behav. Addict..

[29] Brand M., Young K.S., Laier C., Wölfling K., Potenza M.N. (2016). Integrating psychological and neurobiological considerations regarding the development and maintenance of specific Internet-use disorders: An Interaction of Person-Affect-Cognition-Execution (I-PACE) model. Neurosci. Biobehav. Rev..

[30] Brand M., Wegmann E., Stark R., Müller A., Wölfling K., Robbins T.W., Potenza M.N. (2019). The Interaction of Person-Affect-Cognition-Execution (I-PACE) model for addictive behaviors: Update, generalization to addictive behaviors beyond internet-use disorders, and specification of the process character of addictive behaviors. Neurosci. Biobehav. Rev..

[31] Baker T.B., Piper M.E., McCarthy D.E., Majeskie M.R., Fiore M.C. (2004). Addiction motivation reformulated: An affective processing model of negative reinforcement. Psychol. Rev..

[32] Brand M. (2022). Can internet use become addictive?. Science.

[33] Dahlgren C.L., Hage T.W., Wonderlich J.A., Stedal K. (2019). General and eating disorder specific flexibility: Development and validation of the eating disorder flexibility index (EDFLIX) questionnaire. Front. Psychol..

[34] Albertella L., Le Pelley M.E., Chamberlain S.R., Westbrook F., Fontenelle L.F., Segrave R., Lee R., Pearson D., Yücel M. (2019). Reward-related attentional capture is associated with severity of addictive and obsessive–compulsive behaviors. Psychol. Addict. Behav..

[35] Hook R.W., Grant J.E., Ioannidis K., Tiego J., Yücel M., Wilkinson P., Chamberlain S.R. (2021). Trans-diagnostic measurement of impulsivity and compulsivity: A review of self-report tools. Neurosci. Biobehav. Rev..

[36] Lovibond P.F., Lovibond S.H. (1995). The structure of negative emotional states: Comparison of the Depression Anxiety Stress Scales (DASS) with the Beck Depression and Anxiety Inventories. Behav. Res. Ther..

[37] Hayes A.F. (2017). Introduction to Mediation, Moderation, and Conditional Process Analysis: A Regression-Based Approach.

[38] Zorrilla E., Koob G.F. (2019). Impulsivity derived from the dark side: Neurocircuits that contribute to negative urgency. Front. Behav. Neurosci..

[39] Hashimoto N., Nakaaki S., Omori I.M., Fujioi J., Noguchi Y., Murata Y., Sato J., Tatsumi H., Torii K., Mimura M. (2011). Distinct neuropsychological profiles of three major symptom dimensions in obsessive–compulsive disorder. Psychiatry Res..

[40] Wetterneck C.T., Little T.E., Chasson G.S., Smith A.H., Hart J.M., Stanley M.A., Björgvinsson T. (2011). Obsessive–compulsive personality traits: How are they related to OCD severity?. J. Anxiety Disord..

[41] Kitazawa M., Yoshimura M., Murata M., Sato-Fujimoto Y., Hitokoto H., Mimura M., Tsubota K., Kishimoto T. (2018). Associations between problematic Internet use and psychiatric symptoms among university students in Japan. Psychiatry Clin. Neurosci..

[42] Simcharoen S., Pinyopornpanish M., Haoprom P., Kuntawong P., Wongpakaran N., Wongpakaran T. (2018). Prevalence, associated factors and impact of loneliness and interpersonal problems on internet addiction: A study in Chiang Mai medical students. Asian J. Psychiatry.

[43] Shen Y., Meng F., Xu H., Li X., Zhang Y., Huang C., Luo X., Zhang X.Y. (2020). Internet addiction among college students in a Chinese population: Prevalence, correlates, and its relationship with suicide attempts. Depress. Anxiety.

[44] Ordaz S.J., Foran W., Velanova K., Luna B. (2013). Longitudinal growth curves of brain function underlying inhibitory control through adolescence. J. Neurosci..

[45] Ruggieri R.A., Santoro E., De Caro F., Palmieri L., Capunzo M., Venuleo C., Boccia G. (2016). Internet addiction: A prevention action-research intervention. Epidemiol. Biostat. Public Health.

[46] Vondráčková P., Gabrhelik R. (2016). Prevention of Internet addiction: A systematic review. J. Behav. Addict..

[47] Conrod P.J., Castellanos-Ryan N., Strang J. (2010). Brief, personality-targeted coping skills interventions and survival as a non–drug user over a 2-year period during adolescence. Arch. Gen. Psychiatry.

